# Real‐World Evidence on Hospitalization Costs of Pediatric Neoplasm Patients in China: Patterns, Trends, and Associated Factors From a Retrospective Cohort Study

**DOI:** 10.1002/cam4.71635

**Published:** 2026-03-23

**Authors:** Peng Zhang, Bifan Zhu, Linan Wang

**Affiliations:** ^1^ Shanghai University of Traditional Chinese Medicine School of Nursing Pudong District, Shanghai China; ^2^ Shanghai Health Development Research Center Shanghai China; ^3^ Shanghai First Maternity and Infant Hospital School of Medicine, Tongji University Shanghai China

## Abstract

**Background:**

In China, the incidence and burden of pediatric neoplasms have been increasing, contributing to escalating healthcare expenditures and productivity losses, with hospitalization costs constituting a major component of the economic burden. However, evidence on the full spectrum of neoplasm‐related hospitalization costs for pediatric patients remains limited, particularly from real‐world longitudinal studies.

**Methods:**

This retrospective multicenter cohort study (2017–2023) analyzed pediatric hospitalization data in Shanghai by integrating data from two administrative databases. The study included all children aged ≤ 18 years hospitalized with ICD‐10‐coded benign and malignant neoplasms and related complications. Hospitalization costs were discounted to 2023 values, converted to US dollars, and analyzed using descriptive statistics and generalized linear models (GLMs) to identify influencing factors, including socioeconomic, clinical, and hospital‐related variables.

**Results:**

Among 688,131 pediatric hospitalizations, 13,057 (1.91%) were for neoplasms. Neoplasm patients had significantly higher care intensity and hospitalization costs than nonneoplasm patients, with malignant neoplasms incurring the highest median costs. From 2017 to 2023, total costs for neoplasm patients declined by 26.88%, driven by a 52.87% reduction in drug costs, contrasting with rising costs for nonneoplasm patients. Leukemia was the most prevalent condition, while some rare but high‐cost entities, such as secondary malignant neoplasms of the respiratory and digestive organs, were among the most expensive. GLM analysis identified sex, insurance type, pathology, surgical interventions, length of stay, and hospital characteristics as significant cost drivers.

**Conclusion:**

This study provides comprehensive evidence on hospitalization cost patterns, trends, and influencing factors for pediatric neoplasms. It highlights the need for enhanced insurance coverage, early diagnosis and treatment, and equitable resource allocation to reduce disparities.

## 1 Background

Childhood cancer is a significant cause of morbidity and mortality worldwide. It ranks as the second leading cause of noncommunicable deaths among children globally [1], and accounts for 11.5 million global disability‐adjusted life years (DALYs) [2]. Over 80% of childhood cancer cases and 90% of related deaths occur in low‐ and middle‐income countries (LMIC) [3, 4, 5]. As the largest and most populous LMIC, China has been undergoing a rapid epidemiological transition. The burden of childhood neoplasm in China is continuously growing and remains the heaviest [6]. The largest number of incident cases and DALYs of total neoplasm of children, and the highest prevalence of childhood cancer in China, has emerged as a global priority [7].

Meanwhile, childhood neoplasm cases in China pose mounting healthcare costs, crippling financial burdens on families, and alarming productivity losses for a society driven by premature deaths [8, 9]. For China, the total productivity loss caused by these premature deaths was estimated to account for 0.34% ($28 billion) of the country's gross domestic product [10]. For the patients, significant geographic, social, and economic disparities persist [11]. Financially disadvantaged populations face delayed care, inadequate treatment, and heavy economic burdens [12]. These challenges often lead to poverty, psychological distress, and reduced quality of life [13, 14]. Recognizing the importance of Universal Health Coverage (UHC) in enhancing financial risk protection and promoting health equity, China launched an in‐depth healthcare system reform in 2009 and addressed expanding medical insurance coverage [15]. In 2013, eight cancers were included in catastrophic insurance for rural residents, covering at least 70% of inpatient expenses. Since 2015, neoplasm patients have been a priority for drug cost reduction, with centralized volume‐based procurement of neoplasm drugs [16] and national medical insurance negotiations [17].

Despite these efforts to provide financial protection for neoplasm patients, evidence on the costs for pediatric neoplasm patients remains limited. The existing international research primarily focuses on the costs of adult patients rather than pediatric patients and only includes single types of cancer or specific categories [18, 19, 20, 21, 22, 23, 24, 25, 26, 27]. International findings indicate that hospitalization costs account for the largest share of measurable medical expenses [19, 20, 21, 25, 26, 28], although long‐term costs related to complications and survivorship are often unrecorded in hospital‐based data. Nevertheless, there is a lack of comprehensive analyses on the characteristics and hospitalization costs of all neoplasm patients [27]. Focusing on hospitalization costs of pediatric neoplasm patients, scarce data are available for LMICs [3, 18, 29]. To date, limited studies in China have described the costs of malignant solid tumors. These studies used cross‐sectional data from a single‐center [30], or followed up a specific group of children with a subset of neoplasm types (leukemia, solid tumors, and brain tumors) [31, 32]. There is a lack of real‐world studies capturing the full spectrum of neoplasm‐related hospitalization costs, especially based on multicenter, large‐scale cohort studies.

To address this gap, this study includes all pediatric patients diagnosed with benign or malignant neoplasms, as well as those with tumor‐related complications. It utilizes 7 years of retrospective multicenter cohort data to provide updated, comprehensive real‐world evidence on patient characteristics, describe trends and composition of hospitalization costs, identify the most prevalent and high‐cost neoplasms, and examine the key factors influencing these costs. The findings will contribute cost data for health technology assessments, provide real‐world evidence to inform the improvement of health insurance policies, and facilitate the optimization of service delivery and resource allocation for pediatric oncology.

## 2 Methods

### 2.1 Study Design and Data Resource

This study adopts a retrospective cohort design, utilizing hospitalization data from January 1, 2017, to December 31, 2023. The hospitals included were all public hospitals in Shanghai with pediatric inpatient services. Participation was based on data integration with the insurance systems, not on voluntary recruitment. The multicenter data encompassed over this seven‐year period, providing real‐world evidence.

Since 2011, over 95% of China's population has been covered by mandatory social medical insurance schemes, with children enrolled under the Urban and Rural Resident Basic Medical Insurance (URBMI). Additionally, since 1996, children in Shanghai have had access to supplementary coverage through the Children's Hospitalization Mutual Fund (CHMF) [33]. This fund is a public welfare, nonprofit mutual aid initiative designed to mitigate the financial risks due to pediatric hospitalizations and significant outpatient expenses.

Our study integrates data from two complementary administrative databases, the URBMI and the CHMF. This integration enables patient‐level tracking of all hospitalizations and associated costs for pediatric patients across all medical institutions in Shanghai from 2017 to 2023. These two datasets, with consistent and precise standards, were merged to provide comprehensive information and real‐world evidence, forming a solid foundation for robust analysis among pediatric neoplasm patients in the region.

### 2.2 Study Population

The study population consists of all children aged 18 years and under who were hospitalized in Shanghai from 2017 to 2023. Pediatric neoplasm cases were identified based on the International Classification of Diseases Tenth Revision (ICD‐10) codes. For patients with multiple hospitalizations within a year, the diagnosis associated with the highest single hospitalization cost was retained as the primary diagnosis for that year.

The inclusion criteria encompassed: (1) primary diagnoses starting with “C” for malignant neoplasms (C00–C97); (2) primary diagnoses starting with “D” for benign neoplasms (D00–D48); and (3) additional related conditions such as anemia due to neoplasms (D63.0*) and related treatments such as targeted therapy for malignant neoplasms (Z51.801), etc. Exclusion criteria included: (1) cases with missing ICD‐10 codes or diagnoses outside the scope; (2) codes related to conditions before a formal neoplasm diagnosis (e.g., Z03.1 for suspected malignancy and Z12 for neoplasm screening), or cases where the patient's mother has neoplasm as noted in the diagnostic remarks, such as supervision of pregnancy with a history of ovarian malignancy (Z35.802); (3) and codes beginning with “M” for neoplasm morphology, as these are classified under “C” or “D” based on local data standards. This comprehensive approach ensures a precise and standardized identification of pediatric neoplasm patients.

### 2.3 Standardized Hospitalization Cost and Its Structure

In this study, the standardized hospitalization cost was calculated as follows: The individual cost components utilized in the analysis are uniformly defined and standardized across different hospitals, owing to the consistency of reimbursement data. For patients with multiple hospitalization records within a year, the annual hospitalization cost was determined by summing the costs of all individual hospitalizations. To ensure comparability across different years, the annual hospitalization cost per capita was adjusted to 2023 values using the Consumer Price Index for Healthcare to account for inflation and currency fluctuations. Then, the adjusted cost was converted to US dollars using the average exchange rate for 2023 (1 USD = 7.0467 CNY), as the standardized hospitalization cost.

The standardized hospitalization cost for each patient comprised the following costs: (1) Drug costs: Western medicine fees, Chinese medicine fees, etc. (2) Inspection and testing costs: laboratory diagnosis fees, imaging diagnosis fees, clinical diagnosis fees, etc. (3) Medical consumable costs: disposable medical materials fees, etc. (4) Other costs: nursing fees, clinical physical therapy fees, rehabilitation fees, etc. Hence, the hospitalization cost per capita was computed by this formula:

$$\begin{array}{c} {\text{Cost}}_{\text{hospitalization}}={\text{Cost}}_{\text{drug}}+{\text{Cost}}_{\text{inspection and testing}}\\ +{\text{Cost}}_{\text{medical consumable}}+{\text{Cost}}_{\text{other}} \end{array}$$

### 2.4 Factors Influencing Hospitalized Costs

The study considered four categories of potential factors that may influence hospitalization costs. (1) Socioeconomic factors included age, gender, household registration, and type of health insurance. (2) Clinical and therapeutic characteristics encompassed the pathological type of neoplasm and whether surgery was performed. (3) Factors related to healthcare service utilization covered the type of referral, average length of hospital stays (LOS), annual number of hospitalizations, and the proportion of drug costs to total costs. (4) Hospital characteristics, such as hospital level, type, and region, were included.

### 2.5 Statistical Analysis

The study employs descriptive statistical analysis to examine the characteristics, cost variations, and specific neoplasm types among pediatric patients. For categorical variables, frequencies and proportions are reported, while medians and interquartile ranges (IQRs) are used for continuous variables. Due to the nonnormality or heteroscedasticity of the data, the Mann–Whitney *U* test is applied to assess differences in distributions of characteristics and costs between patients with and without neoplasms.

To identify factors influencing hospitalization costs, a generalized linear model (GLM) with a gamma distribution and log link is employed. GLM is suitable for our study because the hospitalization cost data are continuous, strictly positive, and typically right‐skewed. The variables included in the GLM were selected based on relevance to four conceptual domains and availability and completeness within the administrative dataset. Specifically, the study constructed three models, progressively incorporating potential influencing factors: (1) Model 1: included socioeconomic factors and clinical and therapeutic characteristics; (2) Model 2: built upon Model 1 by adding healthcare service utilization factors; (3) Model 3: extended Model 2 by incorporating hospital characteristics.

## 3 Results

### 3.1 Characteristics of the Neoplasm and Nonneoplasm Hospitalized Pediatric Patients

A total of 13,057 neoplasm patients and 675,074 nonneoplasm patients were included in the study, and their characteristics are shown in Table 1. Neoplasm patients accounted for 1.91% of the overall population. Neoplasm patients were older on average, with 37.15% aged 13–18 years, compared to 13.81% in nonneoplasm patients. Boys slightly outnumbered girls among neoplasm patients (50.10% vs. 49.90%), while nonneoplasm patients had a higher male proportion (58.64%). Most patients were registered in Shanghai and covered under the URBMI+CHMF plan, though neoplasm patients had higher proportions with single coverage (URBMI or CHMF). Among neoplasm patients, malignant neoplasms were predominant (56.12%), and 85.91% of neoplasm patients underwent surgery, which was significantly higher than 66.64% among nonneoplasm patients.

**TABLE 1:** Characteristics of neoplasm patients versus nonneoplasm pediatric patients.

Characteristics	Neoplasm patients		Nonneoplasm patients		*p*
	*n*	%	*n*	%	
Total	13,057	100.00	675,074	100.00	
Socioeconomic					
Age*					
≤ 30 days	89	0.68	90,573	13.42	**0.00**
> 30 days to < 1 year	214	1.64	14,391	2.13	
1–4 years	3252	24.91	246,165	36.46	
5–12 years	4651	35.62	230,719	34.18	
13–18years	4851	37.15	93,226	13.81	
Sex*					
Girls	6516	49.90	279,194	41.36	**0.00**
Boys	6541	50.10	395,780	58.64	
Household register					
Shanghai	10,233	78.37	539,825	79.97	**0.00**
Others	2824	21.63	135,249	20.03	
Health insurance type*					
URBMI+CHMF	9196	70.43	533,099	78.97	**0.03**
URBMI	1912	14.64	49,679	7.36	
CHMF	1949	14.93	92,296	13.67	
Clinical and therapeutic					
Pathological type					
Benign	5357	41.03	—	—	
Malignant	7328	56.12	—	—	
Related complications	372	2.85	—	—	
Operation*					
No	1840	14.09	225,199	33.36	**0.00**
Yes	11,217	85.91	449,875	66.64	
Healthcare service utilization					
Referral type*					
Outpatient	6370	48.79	407,173	60.32	**0.00**
Emergency	1134	8.68	109,538	16.23	
Inpatient	2620	20.07	61,172	9.06	
Unknown	2933	22.46	97,191	14.40	
Length of stay, mean (SD)*	21.26	39.80	6.56	10.61	**0.01**
Number of hospitalizations, mean (SD)*	3.25	4.72	1.19	0.79	**0.00**
Proportion of drug costs, mean (SD)*	9.20%	11.28%	11.45%	24.80%	**0.02**
Hospital characteristics					
Hospital level*					
Primary	46	0.35	356	0.05	**0.00**
Secondary	786	6.02	174,185	25.80	
Tertiary	12,225	93.63	500,533	74.14	
Hospital type*					
General hospital	7271	55.69	370,245	54.85	**0.00**
Specialized hospital	5193	39.77	218,483	32.36	
Others	593	4.54	86,346	12.79	
Hospital region*					
Suburb	3922	30.04	303,896	45.02	**0.00**
City center	9135	69.96	371,178	54.98	

Abbreviations: CHMF, the Children's Hospitalization Mutual Fund; URBMI, the Urban and Rural Resident Basic Medical Insurance.

* The symbols in the table represent significant differences between the two groups with *p*‐values < 0.05.

Compared to nonneoplasm patients, neoplasm patients were significantly less often admitted via outpatient services (60.32% vs. 48.79%), experienced longer hospital stays (21.26 days vs. 6.56 days), and had more hospitalizations annually (3.25 vs. 1.19). The proportion of drug costs to total costs of neoplasm patients was significantly lower than that of nonneoplasm patients (9.20% vs. 11.45%). Also, the majority of neoplasm patients were treated in tertiary hospitals (93.63% vs. 74.14%), and more frequently in specialized hospitals (39.77% vs. 32.36%), with a higher concentration in city‐center facilities (69.96% vs. 54.98%).

### 3.2 Trends and Structure of Hospitalization Costs in Neoplasm and Nonneoplasm Hospitalized Pediatric Patients

The median annual hospitalization costs for neoplasm and nonneoplasm patients from 2017 to 2023 are summarized in Table 2. The hospitalization costs for neoplasm patients were significantly higher than those for nonneoplasm patients ($1553.29 vs. $1026.20 in 2023), both in overall terms and across subgroups. The malignant neoplasm patients consistently incurred the highest hospitalization costs, which were significantly higher than those for patients with related complications and benign neoplasms ($2162.81 vs. $1796.73 and $1281.02 in 2023).

**TABLE 2:** Trends of annual hospitalization costs 2017–2023: Neoplasm versus nonneoplasm pediatric patients (Median, $).

Year	Neoplasm patients				Nonneoplasm patients
	Benign	Malignant	Other complications	Total	
2017	1452.75	3138.01	2299.13	2124.01	779.88
2018	1361.01	2403.70	2536.29	1894.27	822.25
2019	1540.79	2434.63	1521.37	1974.12	889.83
2020	1375.35	2767.87	1664.61	1952.07	1092.48
2021	1515.24	3099.90	1913.44	2093.88	1203.72
2022	1507.46	3100.22	1520.14	2028.45	1221.40
2023	1281.02	2162.81	1796.73	1553.29	1026.20

In terms of trends, from 2017 to 2023, the hospitalization costs for neoplasm patients fluctuated but showed an overall decline of 26.88% (from $2124.01 to $1553.29), while for nonneoplasm patients, it demonstrated an upward trend, with costs increasing by 31.55% (from $779.88 to $1026.20). Among the neoplasm patients, the hospitalization costs for malignant neoplasm patients exhibited a steady decline, dropping by 31.08% (from $3138.01 to $2162.81). Following this, the median costs for patients with other complications and with benign neoplasms decreased by 21.9% (from $2299.13 to $1796.73) and 11.8% (from $1452.75 to $1281.02), respectively.

**FIGURE 1:**
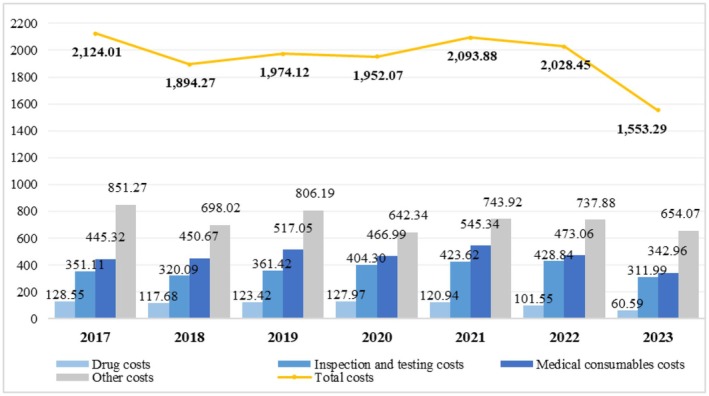
Trends and composition of costs for neoplasm patients 2017–2023 (Median, $).

The structure of hospitalization costs among neoplasm pediatric patients is shown in Figure 1. For overall cost structure, among the major components, medical consumable costs were consistently higher than those of inspection and testing, followed by drugs. In 2017, consumable costs accounted for 20.97% ($445.32) of the total costs, inspection and testing costs represented 16.53% ($351.11), and drug costs made up 6.05% ($128.55). By 2023, the proportion of consumable costs remained higher at 22.08% ($342.96). Other costs consistently accounted for the largest share in all years, such as 40.08% ($851.27) in 2017 and 42.11% ($654.07) in 2023.

From 2017 to 2023, with the total costs decreasing by 26.87%, all components showed a downward trend. Among these, drug costs experienced the largest decline at 52.87% (from $128.55 to $60.59), followed by other costs at 23.17% (from $851.27 to $402.54), and medical consumable costs, which decreased by 22.99% (from $445.32 to $342.96). The smallest reduction was observed in inspection and testing costs, with an 11.14% decrease (from $351.11 to $311.99).

### 3.3 Most Prevalent Diseases and Most Costly Diseases Among Pediatric Patients With Neoplasm

The top 10 most prevalent diseases and the top 10 most costly diseases among children with neoplasm are listed in Table 3. The results reveal that among the top 10 most prevalent neoplasms, malignant and benign neoplasms each account for half. The most common conditions in children are leukemia (other and unspecified cell type leukemia, C95: 11.3%, and myeloid leukemia, C92: 2.95%), followed by hemangioma and lymphangioma (D18: 8.88%) and other and unspecified benign neoplasms (D36: 5.51%). Other frequently occurring malignant neoplasms include malignant neoplasms of the bones and articular cartilage of the limbs (C40: 5.09%), other and unspecified malignant neoplasms (C76: 3.83%), and malignant neoplasms of the brain (C71: 3.66%). Meanwhile, the median costs of malignant neoplasms are generally higher than those of benign neoplasms.

**TABLE 3:** Most prevalent and most costly diseases among neoplasm pediatric patients 2017–2023.

Disease		Prevalence and costs			
Most prevalent diseases		ICD10	*n*	%	Median, $
1	Leukemia, other and unspecified cell type	C95	1475	11.30	6704.60
2	Hemangioma and lymphangioma	D18	1160	8.88	1522.40
3	Benign neoplasm of other and unspecified sites	D36	719	5.51	1207.05
4	Malignant neoplasm of bone and articular cartilage	C40	664	5.09	4095.03
5	Melanocytic nevi	D22	579	4.43	938.86
6	Malignant neoplasm of other and unspecified sites	C76	500	3.83	1172.62
7	Benign neoplasm of breast	D24	480	3.68	908.63
8	Malignant neoplasm of brain	C71	478	3.66	10,880.09
9	Benign neoplasm of bone and articular cartilage	D16	409	3.13	2423.37
10	Myeloid leukemia	C92	385	2.95	3321.76

Most costly diseases		ICD10	Median, $	*n*	%
1	Secondary malignant neoplasm of respiratory and digestive organs	C78	16,796.03	16	0.12
2	Neoplasm of uncertain or unknown behavior of endocrine glands	D44	11,214.85	36	0.28
3	Malignant neoplasm of retroperitoneum and peritoneum	C48	11,040.77	70	0.54
4	Malignant neoplasm of brain	C71	10,880.09	480	3.68
5	Benign neoplasm of brain and other parts of central nervous system	D33	10,590.86	28	0.21
6	Neoplasm of uncertain or unknown behavior of brain and central nervous system	D43	10,484.08	72	0.55
7	Malignant neoplasm of pancreas	C25	9852.26	11	0.08
8	Follicular lymphoma	C83	9684.71	50	0.38
9	Neoplasm of uncertain or unknown behavior of mouth and digestive organs	D37	8182.97	37	0.28
10	Lymphoid leukemia	C91	8171.55	122	0.93

Abbreviation: ICD, international classification of diseases.

Among the top 10 most costly neoplasms, six were malignant. The highest median annual hospitalization cost incurred among patients with secondary malignant neoplasms of the respiratory and digestive organs (C78: $16,796.03), followed by benign neoplasms of uncertain or unknown behavior of endocrine glands (D44: $11,214.85) and malignant neoplasms of the retroperitoneum and peritoneum (C48: $11,040.77). Malignant neoplasms of the brain were the only condition appearing in both the most prevalent and most costly categories (C71: $10,880.09). Furthermore, neoplasms such as benign neoplasms of the brain and other parts of the central nervous system (D33: $10,590.86) and neoplasms of uncertain or unknown behavior of the central nervous system (D43: $10,484.08) also incurred high costs. Other malignant neoplasms with high costs included malignant neoplasms of the pancreas (C25: $9852.26), follicular lymphoma (C83: $9684.71), and lymphoid leukemia (C91: $8171.55).

### 3.4 Influencing Factors of the Hospitalization Cost Among Pediatric Neoplasm Patients

The results of three multivariate GLMs analyzing the association between various factors with healthcare costs are presented in Table 4. As more factors were included in the models, additional influencing factors were identified from Model 1 to Model 3. While the coefficients showed slight variations, their significance remained largely robust.

**TABLE 4:** Factors influencing hospitalized costs for pediatric neoplasm patients: GLM results across models.

Characteristics	Model 1		Model 2		Model 3	
	Coefficient	*p*	Coefficient	*p*	Coefficient	*p*
Socioeconomic						
Age ref. ≤ 30 days						
> 30 days to < 1 year	−0.08	0.75	−0.13	0.36	−0.08	0.55
1–4 years	0.07	0.75	−0.20	0.09	−0.15	0.22
5–12 years	−0.05	0.82	−0.30	0.10	−0.25	0.14
13–18 years	−0.04	0.87	−0.21	0.07	−0.17	0.15
Sex ref. Girls						
Boys	0.21	0.00	0.05	0.02	0.04	**0.04**
Household register ref. Shanghai						
Others	0.23	0.00	0.15	0.00	0.16	**0.00**
Health insurance type ref. URBMI+CHMF						
URBMI	0.48	0.00	0.34	0.00	0.50	**0.00**
CHMF	0.22	0.00	0.23	0.00	0.23	**0.00**
Clinical and therapeutic						
Pathological type ref. Benign						
Malignant	1.11	0.00	0.35	0.00	0.32	**0.00**
Other complications	0.84	0.00	0.08	0.22	0.06	0.36
Operation ref. No						
Yes	0.98	0.00	0.53	0.00	0.52	**0.00**
Healthcare service utilization						
Referral type ref. outpatient						
Emergency			0.09	0.01	0.12	**0.00**
Inpatient			0.28	0.00	0.17	**0.00**
Length of stay			0.02	0.00	0.03	**0.00**
Number of hospitalizations			0.00	0.68	0.00	0.29
Proportion of drug costs			0.80	0.00	0.93	**0.00**
Hospital characteristics						
Hospital level ref. primary						
Secondary					0.11	**0.04**
Tertiary					0.68	**0.00**
Hospital type ref. General hospital						
Specialized hospital					−0.26	**0.00**
Others					−0.14	0.20
Hospital region ref. suburb						
City center					0.02	0.40
_cons	7.04	0.00	7.08	0.00	7.34	**0.00**

Abbreviations: _cons, consistency; CHMF, the Children's Hospitalization Mutual Fund; URBMI, the Urban and Rural Resident Basic Medical Insurance.

Overall, common factors significantly associated with higher inpatient costs included being male (compared to female, *p* = 0.04), having a household registration in Shanghai (compared to nonlocal, *p* < 0.001), and being enrolled in a single health insurance scheme (compared to dual enrollment in URBMI and CHMF, *p* < 0.001). Compared to benign neoplasms, malignant neoplasms were associated with significantly higher costs (*p* < 0.001), and patients undergoing surgery also incurred significantly higher expenses (*p* < 0.001). In terms of healthcare service utilization, patients admitted from emergency or inpatient referral had significantly higher costs compared to those admitted via outpatient referral (*p* < 0.001). Longer average length of stay and higher drug cost proportion were significantly associated with higher costs (*p* < 0.001), whereas the number of hospitalizations was not significant. Regarding hospital characteristics, secondary (*p* = 0.04) and tertiary (*p* < 0.001) hospitals had higher costs compared to primary hospitals, and general hospitals were associated with higher costs compared to specialized hospitals (*p* < 0.001). However, whether the hospital was located in the city center had no significant impact.

## 4 Discussion

By leveraging 7 years of multicenter retrospective cohort data, this study focuses on all hospitalized pediatric patients diagnosed with benign neoplasms, malignant neoplasms, and related complications in Shanghai, China, to generate real‐world evidence on their hospitalization costs. The study systematically examined individual socioeconomic and clinical characteristics, patterns of healthcare utilization, and the characteristics of inpatient hospitals; mapped the trends and structure of their hospitalization costs; identified the most common and costly specific diseases; and determined the factors influencing these costs.

The findings indicate that pediatric neoplasm patients accounted for a small proportion (1.91%) of the hospitalized population but incurred significantly higher hospitalization costs compared to nonneoplasm patients. This proportion is slightly higher than the percentage of children with neoplasm under Medicaid in the United States (less than 1%) [25], which may be related to the broader accessibility resulting from China's higher health insurance coverage rate. The declining trend in hospitalization costs for pediatric neoplasm patients observed in our study aligns with findings from other parts of China. A recent study based on data from a tertiary hospital in mainland China reported a similar downward trend in inpatient expenses among children with malignant solid tumors, attributing the change to the expanded coverage of anticancer drugs under national health insurance and increased access to targeted therapies through bulk procurement programs [31, 32].

Significant differences in characteristics exist between neoplasm and nonneoplasm pediatric patients. This slight male predominance is consistent with epidemiological evidence showing a higher incidence of certain pediatric cancers—such as acute lymphoblastic leukemia, medulloblastoma, and lymphomas—among boys, which likely contributes to the observed gender distribution in our cohort. Higher intensity of care is required for neoplasm management, including frequent hospitalizations, prolonged hospital stays, and specialized treatments such as surgery and targeted therapies [19, 29]. They also utilized these services in tertiary and general hospitals in the city center, which was also observed in another study [31]. As a result, in 2023, the median annual hospitalization cost for neoplasm patients was $1553.29, which was approximately 51.3% higher than that of nonneoplasm patients. This significant cost disparity reflects the exceptionally high healthcare service needs and the heavy disease burden, with the potential concern of health inequity among these vulnerable neoplasm patients [19, 27].

Encouragingly, the hospitalization costs for all neoplasm patients showed a decline of 26.88% from 2017 to 2023, although the overall costs for nonneoplasm patients increased. In contrast, the upward trend in hospitalization costs for nonneoplasm pediatric patients may reflect a combination of factors. Beyond the expanded benefit packages associated with China's universal health coverage [34], the increased utilization of newly approved therapies—such as gene therapy, monoclonal antibodies, and advanced nutritional and supportive treatments—has contributed to higher treatment intensity and expenditures. Many of these conditions, previously managed conservatively, are now treated more aggressively due to advancements in diagnostics and therapeutic availability, which may partly explain the continuous rise in inpatient costs for this group. The downward trend in costs for neoplasm patients has also been confirmed in similar studies of children with leukemia in China [32]. Considering the cost structure among neoplasm patients, the most significant reduction of 52.87% in costs was observed in the drug expenses, which may be a key driver of the overall cost decline. This reduction is closely linked to a series of pharmaceutical reform policies implemented in China since 2015, including centralized volume‐based procurement of neoplasm drugs [16] and national medical insurance negotiations [17]. These policies have significantly improved access to medicines and reduced the financial burden on patients [35, 36, 37]. Also, the lower proportion of drug costs among neoplasm patients indicates a shift in healthcare costs associated with neoplasm treatment, likely influenced by changes in medical practices, therapies, and inflationary factors, which is also influenced by the realignment of the incentives for providers in China [38]. Nevertheless, neoplasm patients consistently face higher costs, which underscores the need for targeted policy interventions to continuously decrease their disease burden and costs [39].

Notably, malignant neoplasm patients had the highest hospitalization costs, consistently exceeding those for patients with benign neoplasms or related complications. This is consistent with the resource‐intensive nature of malignant neoplasm care, which often involves complex diagnostic and therapeutic interventions. Specifically, malignant neoplasms of the brain ranked in the top 10 for both the most common and the most expensive conditions, and patients with this condition always incur treatment abandonment due to financial constraints, travel distances, along with prolonged and complex treatment courses and uncertainties in outcomes [40]. Meanwhile, different subtypes of leukemia remain among the most prevalent and economically burdensome diseases for hospitalized children, consistent with findings from international studies [18, 20, 26, 29, 31]. In addition, benign neoplasms of uncertain or dynamic nature in the brain and central nervous system, endocrine glands, and oral and digestive organs also show relatively high median costs, highlighting the importance of early diagnosis to reduce the need for costly, late‐stage interventions [41].

The results of GLM highlight that male sex, single insurance coverage (URBMI or CIMMF), malignant tumor pathology, undergoing surgery, emergency or inpatient referral, longer length of hospital stay, higher proportion of drug costs, and treatment in secondary or tertiary general hospitals were all significantly associated with increased hospitalization costs. Malignant neoplasms, surgical treatment, tertiary care in general hospitals, and longer hospital stays were particularly associated with higher expenditures. These factors partly reflect the high severity of illness among neoplasm patients, the complexity of treatment regimens, and the costly and intensive medical resources utilized, all of which contribute to higher expenses [31]. Particular attention should be paid to resource‐related factors associated with patients, such as neoplasm patients from outside Shanghai or covered by a single insurance plan incurring disproportionately higher costs [21, 25]. These findings suggest that despite Shanghai's well‐established insurance schemes, socioeconomic and geographical factors remain barriers to equitable access to care and disparities were observed in economic burdens [42].

The study holds significant policy implications for reducing the economic burden of pediatric neoplasms and improving healthcare equity. Firstly, the high costs highlight the need to expand insurance coverage and enhance reimbursement policies to provide better financial protection for families [42]. The observed decline in medication costs for neoplasm patients underscores the success of drug procurement reforms, such as volume‐based procurement and national insurance negotiations, which should be sustained and expanded. Secondly, focusing on the major conditions with high prevalence and costs, promoting early diagnosis and timely treatment through screening programs, and optimizing inpatient care pathways could help reduce costs and improve outcomes [41]. Additionally, addressing disparities in resource allocation and optimizing service delivery across regions and hospital types is crucial. Special attention should also be given to high‐cost subgroups, such as those with rare or complex neoplasms or with a single insurance scheme, to mitigate inequities and ensure better access to care. Although data identifying high‐cost subgroups (e.g., male pediatric patients with malignancies) could potentially be misused by private insurers to adjust premiums, this concern is less applicable in the Chinese context, where basic medical insurance is publicly funded and centrally managed. The primary objective of the basic medical insurance in China remains equitable coverage and risk pooling and the results provide valuable real‐world evidence to support targeted financial protection strategies, such as dynamic adjustment of reimbursement policies, early inclusion of high‐cost diseases in catastrophic insurance, and regional resource reallocation based on patient distribution and burden. Responsible use of such data by government, public insurance systems, and private insurance companies is required to enhance efficiency and equity, rather than exacerbate disparities.

This study has some limitations. First, due to data limitations, studies in China often face challenges in obtaining outpatient visits and cost data for individual patients, making it difficult to link these with inpatient costs. Previous studies have shown that most expenses for neoplasm patients occur during hospitalization [19]. By focusing on inpatient costs, we provide real‐world evidence of the primary cost patterns for these patients. Future research should extend beyond hospitalization costs to include outpatient services, nursing, rehabilitation, and other comprehensive expenditures, as well as caregivers' time costs and intangible burdens, in order to reflect the full life‐cycle economic burden of pediatric neoplasm patients. Second, it relied on administrative data, which may not capture clinical outcomes or patient‐reported experiences. Future research will aim to overcome data barriers and integrate survey methods to explore the long‐term impact of pharmaceutical and insurance reforms on treatment access, financial protection, and health outcomes, particularly for high‐cost or rare pediatric cancers. Third, the generalizability of findings to other regions in China may be limited due to the unique healthcare infrastructure and socioeconomic context of Shanghai. These findings may reflect trends in other urban areas of China and LMICs undergoing similar insurance reforms, though caution is needed when generalizing to rural or underserved settings. Nevertheless, this study seeks to update the cost profile of pediatric neoplasm patients, providing real‐world data to inform subsequent health technology assessments, drug pricing, and policy‐making processes.

## 5 Conclusion

This study provides real‐world evidence on the characteristics, hospitalization cost trends and structure, and most prevalent and costly diseases of pediatric neoplasm patients in China, and identifies the factors influencing costs. The results highlight significant disparities in hospitalization costs of neoplasm patients, and their substantial healthcare needs and utilization, resulting in a heavy economic burden, particularly for those with malignant neoplasms and limited resources. The findings underscore the need for targeted policy interventions to improve health coverage, promote early diagnosis and treatment, optimize service delivery, and address healthcare inequities among pediatric neoplasm patients.

## Author Contributions

P.Z.: conceptualization, software, methodology, formal analysis, writing – original draft, writing – review and editing, visualization. B.Z.: conceptualization, data curation, investigation, funding acquisition, writing – review and editing. L.W.: validation, supervision, writing – review and editing, project administration, funding acquisition, resources.

## Funding

This work was supported by Science and Technology Commission of Shanghai Municipality, No. 23692109000, No. 23YF1440900.

## Ethics Statement

This study was approved by the Ethics Committee of the Shanghai Health Development Research Center (Approval number: 2024001). All patient data were anonymized before analysis. Since the data we used came from anonymized and secondary databases, human participants were not directly involved in the study, and the informed consent was exempted. All methods were carried out in accordance with relevant guidelines and regulations (declarations of Helsinki, 1996). The consent process incorporates statements about the use of anonymized data for publication.

## Conflicts of Interest

The authors declare no conflicts of interest.

## Supporting information


**Data S1:** cam471635‐sup‐0001‐DataS1.pdf.

## Data Availability

The datasets analyzed during the current study are not publicly available due to local data governance regulations but are available from the corresponding author on reasonable request and with permission from the data providers.
