# Supplementary material for: Real‐World Evidence on Hospitalization Costs of Pediatric Neoplasm Patients in China: Patterns, Trends, and Associated Factors From a Retrospective Cohort Study
Source: Cancer Med. 2026 Mar 23;15(3):e71635. doi: 10.1002/cam4.71635 (PMC13140997; doi:10.1002/cam4.71635)
Supplement: Supplementary file 1 — Data S1: cam471635‐sup‐0001‐DataS1.pdf. [file CAM4-15-e71635-s001.pdf]

Ethics Review Approval Letter

Statement: The ethics committee of this institution is organized and worked in accordance with the relevant regulations of the National Health Commission and CFDA, and its review and work process are not affected by any organization or individual other than the ethics committee of this institution.

Approval Number: 2024001

Review date/Review meeting place	22nd May 2024
Research project name	Shanghai Children's inpatient Fund fee standard for 2024 academic year
Review documents	Preliminary Review（Preliminary decision at 22nd,May,2024）<br>Second Review（Second Review decision at13th,June,2024）
Applicant	Duo Chen
Research institution	Shanghai Health Development Research Center (Shanghai Health Information Center)
Main researcher	Chunlin Jin, Linan Wang, Duo Chen, Bifan Zhu, Xiaohui Hou
Ethical review	☐Meeting review ☑Quick review
Review comment	1. After review by ethics committee of this institution, the study was agreed.<br>Comments and suggestions: ☑YES ☐NO<br>2. Annual/regular follow-up review of the implementation process of the study by the ethics committee:<br>☐YES ☑NO<br>The frequency of review is from the date of approval of the study:<br>☐3 months ☐6 months ☐12 months<br>3. The ethics committee of this institution has the right to change the frequency of annual / regular follow-up reviews based on actual progress.<br>4. If the project is not started within one year from the date of approval, the approval will automatically become invalid.

The signature of the director or deputy director: Chunlin Jin

Institution Ethics Committee (Seal)

date: 2024.6.13

Note: (please read carefully)

1. The project approved by the ethics committee of this institution is biomedical research involving the human body, and the research must be carried out in strict accordance with the latest version of the approved research plan and informed

consent, and in accordance with the relevant domestic regulatory guidelines.

2. Any content that involves the export of human genetic resources or must be subject to special approval by the relevant department in accordance with national regulations , must be reported to and approved by the relevant department before the project is executed.

3. This approval document may be used for reference by other central ethics committees. If there are different opinions about review, please communicate with the ethics committee of this institution in time.

4. Any modification of the approved research plan, informed consent and other materials, and the replacement of the main resercher and so on, must be notified to the ethics committee of the institution in time for additionally review and execute after approval.

5. Serious adverse events and unexpected events that affect the risk-benefit ratio of the research must be reported to the ethics committee of the institution in time.

6. According to the opinion of ethics committee on the frequency of annual follow-up review, regardless of whether the research is started or not, please submit an application for annual /regular follow-up review 1 month before the date of the annual follow-up review.

7. It is necessary to report to the ethics committee for review in case of non-compliance or violation.

8. Suspending or early terminating clinical research, please inform the ethics committee in time.

9. Once the research is done, a concluding report must be submitted for review by the ethics committee.

Address: No. 1477 Beijing West Road, Jingan District, Shanghai; Postcode: 200000; Phone: 021-22121863
